# Report of a Case of Sciatica

**Published:** 1873-09-01

**Authors:** T. D. Washburn


### REPORT OF A CASE OF SCIATICA.

READ BEFORE THE MONTGOMERY CO. MEDICAL SOCIETY, BY T. D. WASHBURN, M.D.

On the 7th of April last, was called to see Mr. Abner Short, a farmer, aged 60; well developed; average vitality; nervo-sanguine temperament; good habits, and good general health. Eight years before had suffered with such severe pain in the hip as to call a doctor, and was relieved with a few doses of morphia. A month since he had trouble with his kidneys and bladder — pain, frequent micturitions, with sediment and turbid urine, which he thinks was relieved by homœopathic treatment. For some weeks now pain has been gradually increasing along the course of the sciatic — more intense externally and at night — painful to sit, lie or walk on the affected limb (the right). Having rubbed with various liniments, he tried a blister a week since, without relief.

I find him now with hot skin, coated tongue, constipated bowels, bad breath, headache, high colored urine, and much pain in region of hip; no appetite; pulse 75; thirst; also pain over right hypogastrium and bowels and testicle, the latter swollen, indicating effusion. I gave at once *three* comp. cath. pills, and left a solution of antimony, potassa nit., and a small portion of morphia, to be given in three hours, and to repeat every two hours until nausea or perspiration.

April 9th. Found my patient much relieved. Fever subsided, bowels well moved, skin and kidneys acting, but pain still present. I now applied a blister (2x4) over the pain, just back of trochanter; order it kept open and dressed with morphia acet., gr. i. (rubbed up with sugar), daily; take iod. potassa, grs. v., tinct. aconite, gtts. v., three times a day.

April 13th. Found the iodide had produced its characteristic eruption, and the aconite had partially controlled the pain; but still patient at times suffers much. Appetite remains fair.

℞.—Quinia,
Cinchona-quinia, a a. grs. iiss.
Morphia sulph., gr. is.—M.

Of which take *one* three times a day.

April 16th. While the morphia was taken, the pain was partially controlled. I now added prussiate ferri to my tonic powders, and gave my morphia combined with fluid ext. valerian, in increased doses. Occasionally compound cath. pills (*two*) are used to keep the bowels open. Urine remains clear and plenty; skin acts well; condition generally normal; not restricted in his diet.

April 20th. Pain persists, but inclined to be more severe about the knee; had a blister placed posteriorly two inches above, and directed to be dressed *twice* a day with a gr. of morphia acet.; withdrew the anodyne internally, also the tonics, and gave spirits of turpentine ʒ i., in mucilage, three times a day, alternating with bromide of potassa, grs. xv. each dose.

April 23d. About this time, finding no satisfactory result from the treatment, I began the use of the battery, local and general galvanism, for fifteen or twenty minutes, twice a day. This gave some relief, but the effect did not seem to last long, so I was compelled to leave ½ gr. powders of morphia, to be given when the pain was severe.

I now gave:

℞.—Tinct. ferri chlor., gtts. xx.

Fluid ext. ergot, ʒ ss.—M.

To be taken before each meal.

Blister having healed, I left strong liniment of aconite, ammonia and turpentine, to apply where pain was worst.

April 27th. Paroxysms of pain occur during the latter part of the night, but are not periodic; the tonics do not control them; nothing but an increased and continued use of morphia gives relief. To-day I put him on wine of colchicum, ʒ ss., three times a day, and chloral, gr. xx., at bed-time.

April 29th. Paroxysms of pain at night continue, sometimes every night, then an omission of one or two. Now and then these paroxysms, from their severity, seem to deprive him of consciousness, and for a few minutes he wanders.

These three weeks he has been confined to his bed, barely able, with the help of a rope suspended from the ceiling, to raise himself or turn over.

The weather has been cold, damp and changeable. When pain was intense, cloths wrung out of boiling hot water have been applied, giving partial relief. Pain in the testicle, which has continued to increase in size, has been modified by the free use of tobacco fomentations. To be sure as to what was the matter, passed an exploring needle, and drew off two tablespoonsful of serous water.

Continued colchicum, and alternate with tinct. guiac, ʒ doses.

May 4th. Remains much the same; has not apparently lost in flesh or strength, but suffering has been little modified by treatment. The battery appears to have changed the *character* of the pains somewhat, making them less paroxysmal, and not so difficult to bear; less periodic and neuralgic, and more continuous. Withdrew all remedies except aconite and mur. ammonia, leaving some morphia powders for emergencies.

May 6th. I find him suffering so much that I have withdrawn everything, and used the hypodermic injection of ⅜ gr. morphia in the right arm (which I should have done sooner had the patient been near town). In ten minutes the pain was gone, and he was free from suffering for near twenty-four hours, when I repeated the same dose. A teaspoonful of salts was sufficient to move the bowels. I now urge him to begin to use his strength, sit up, and walk across the room. In a week he was about the place, steadily gaining, able to lie on the affected side, and begins to ride out.

After a few days I gave the charge of the injection to a neighbor of his; and soon after one of his own family took charge of it; and from that time to the present, more than six weeks, the injection has been used, with *one* or *two* exceptions, daily. His arm is well scarred. I gave one over the seat of pain, in the hip, but it did not act as promptly as in the arms; have given in both arms. He *thinks* his fingers *partially* paralyzed from the use of it; *no other* inconvenience.

When I attended the State Medical Society, at Bloomington, I expected to meet some one who could pilot me out of this formidable case—perhaps with a specific, or other reliable remedy—but I found none even sanguine of a speedy cure. I intended to have buttonholed Dr. Prince, of Jacksonville, who was giving special attention to galvanism, but our time was so much engrossed I failed to do it. I have endeavored faithfully to consult authorities — Tweedie, Wood, Flint, Burnet, Graves, Marshall, Hall, Brown, Sequard, Hafeland, Niemeyer, Braithwaite; *Medical and Surgical Electricity*, by Beard and Rockwell; but none can satisfactorily give the pathology, and hence fail to meet the remedy.

Prof. Andrews, of Chicago, advised to make inspection of the *rectum*, as some cases had been so masked that the real cause, a tumor in the rectum, had been overlooked; but the whole history of the case, and personal inspection, forbid such an hypothesis. If the kidneys were not performing their functions so well, I might look there possibly for some key to this continued pain; but I think both these organs innocent. The latest authority, in which the writer speaks of his own experience in this painful disease, is Dr. Lawson, in the *Dublin Journal of Medical Science*, June, 1872. Also, in *Braithwaite*, part 66, He admits that he is exposed to the criticism of treating a symptom, but adds that the symptom (pain) is the whole of it; by arresting that we remove the trouble. He is of the opinion we know nothing of the pathology of sciatica; and I am partly confirmed in this view, by the testimony of others, in the general exception they make of *sciatica*, in treating other neuralgias.

As to adjuvants, Dr. Lawson has found cod liver oil, quassia and iron invaluable. He thinks well of alcohol, in bad cases and impaired vitality, in the form of whisky or brandy; but discards the use of beer, wine, tea and coffee. He says the hypodermic injection may be continued for months; nausea is less likely to occur if the stomach has food. *

Next, perhaps, in as positive terms, is Dr. Blackiston, in *Medical Times and Gazette*, (Dublin), 1855, in which he quotes 83 cases treated with the blister and acet. morphia, as reported in the early part of this paper. But it certainly failed to give more than temporary relief in this case (though carried out to the letter). Next in power to this, and well worth a trial, is the use of a small metal disc, heated in a spirit-lamp and passed rapidly over the painful part, almost blistering by its intense heat, but generally only leaving some redness, or slightly raising the cuticle in spots.

Indian hemp, gelsemium, belladonna, croton oil, vapor baths, etc., have had their

* Since this paper was read, I find, in *The Clinic*, of July 5th, a case reported by Dr. Bartholow, in which 2 grs. of morphia and 1-96th of atropia, were injected, for more than two years, daily. They were gradually diminished, in the course of six months, without jar to the nervous system. He also states that, "One year has now passed since they were discontinued, and no difficulty or embarrassments of any kind has been experienced."

advocates. Alteratives, antimony, tonics, and opium were frequently the heroic remedies. But when your patient is on the verge of distraction, wondering whether there is *any* virtue in medicine; when one is just ready to swear, or to cry, it is about time the doctor was mustering his forces as though he meant business; and the agency most likely to please the patient is the hypodermic injection of morphia.

The solution I used was grs. xxxii. to the oz. of distilled water. There is another agent, whose laws are not yet *well* defined, which I believe may be found useful—*galvanism*. As a nerve tonic, possibly a curative, in this case, I think it would be well to try the compound phophorus pill, or the iodoform and iron.

It seems desirable to make a *change;* the morphine habit is a bad one, and yet he suffers without it. What substitute can we make? I have advised the general withdrawal, by giving a drop less every other day.

I have hastily prepared this paper, and present the patient for your careful inspection and counsel. *

* *Aug. 6th.* Some three or four weeks after using the exploring needle, the sac-head again filled, and was giving uneasiness and pain; so I used a larger exploring needle, and drew off some three ounces, and immediately strapped the testicle as firmly as possible Some hardening and acute inflammation followed, and to my surprise, a *radical* cure had been established.

